# Asymmetric cortical activation in healthy and hemiplegic individuals during walking: A functional near-infrared spectroscopy neuroimaging study

**DOI:** 10.3389/fneur.2022.1044982

**Published:** 2023-01-25

**Authors:** Xiaokuo He, Lei Lei, Guo Yu, Xin Lin, Qianqian Sun, Shanjia Chen

**Affiliations:** ^1^Department of Rehabilitative Medicine, Fifth Hospital of Xiamen, Xiamen, China; ^2^Department of Rehabilitative Medicine, Xiangyang Central Hospital, Xiangyang, Hubei, China; ^3^Department of Rehabilitative Medicine, The First Affiliated Hospital of Xiamen University, Xiamen, China

**Keywords:** stroke, hemiplegia, gait, treadmill walking, functional electrical stimulation

## Abstract

**Background:**

This study investigated the cortical activation mechanism underlying locomotor control during healthy and hemiplegic walking.

**Methods:**

A total of eight healthy individuals with right leg dominance (male patients, 75%; mean age, 40.06 ± 4.53 years) and six post-stroke patients with right hemiplegia (male patients, 86%; mean age, 44.41 ± 7.23 years; disease course, 5.21 ± 2.63 months) completed a walking task at a treadmill speed of 2 km/h and a functional electrical stimulation (FES)-assisted walking task, respectively. Functional near-infrared spectroscopy (fNIRS) was used to detect hemodynamic changes in neuronal activity in the bilateral sensorimotor cortex (SMC), supplementary motor area (SMA), and premotor cortex (PMC).

**Results:**

fNIRS cortical mapping showed more SMC-PMC-SMA locomotor network activation during hemiplegic walking than during healthy gait. Furthermore, more SMA and PMC activation in the affected hemisphere was observed during the FES-assisted hemiplegic walking task than during the non-FES-assisted task. The laterality index indicated asymmetric cortical activation during hemiplegic gait, with relatively greater activation in the unaffected (right) hemisphere during hemiplegic gait than during healthy walking. During hemiplegic walking, the SMC and SMA were predominantly activated in the unaffected hemisphere, whereas the PMC was predominantly activated in the affected hemisphere. No significant differences in the laterality index were noted between the other groups and regions (*p* > 0.05).

**Conclusion:**

An important feature of asymmetric cortical activation was found in patients with post-stroke during the walking process, which was the recruitment of more SMC-SMA-PMC activation than in healthy individuals. Interestingly, there was no significant lateralized activation during hemiplegic walking with FES assistance, which would seem to indicate that FES may help hemiplegic walking recover the balance in cortical activation. These results, which are worth verifying through additional research, suggest that FES used as a potential therapeutic strategy may play an important role in motor recovery after stroke.

## Introduction

Stroke is one of the leading causes of disability in adults worldwide. Although most patients can recover their walking ability after rehabilitation treatment, more than 50% of hemiplegic patients with post-stroke in the community cannot walk independently (1). The central pattern generators in the spinal cord play an important role in the generation of rhythmic and repetitive locomotor patterns *via* supraspinal regulation of cerebral neural networks; nonetheless, the neural substrates and associated neurophysiological mechanisms underlying dynamic locomotor control remain elusive (2, 3). To date, the cortical activation mechanism underlying locomotor control during dynamic walking in stroke patients with hemiplegia and healthy individuals have not yet been elucidated. Functional magnetic resonance imaging, which is characterized by high spatial resolution and low temporal resolution, is used to probe cortical activation patterns during finger, ankle, and knee movements; however, it is ill-suited for studying larger locomotor activities, such as walking, due to inherent motion artifacts and structural constraints (4). Positron emission tomography requires the injection of a radioactive tracer and is unsuitable for repeated measurements (5). Electroencephalography (EEG) has high temporal resolution but low spatial resolution, leading to long preparation times and easy human interference (6). Functional near-infrared spectroscopy (fNIRS) imaging technology is not only a good tool for assessing movements, such as walking and postural transitions, but also an effective tool for examining brain activity patterns during real-world activities (7). fNIRS can detect hemoglobin (Hb) oxygenation during human gait and has been utilized as a signal marker of neural substrates underpinning locomotor control in healthy adults (8) and patients with hemiparetic stroke (3).

Treadmill walking (TW) can provide safe, intensive, and task-oriented rehabilitation for patients with dyskinesia after a stroke and is widely used for clinical gait rehabilitation training (9). For the first purpose of our research, we used fNIRS to monitor cortical blood oxygen changes during healthy and hemiplegic walking and to explore differences in brain activation between normal and hemiplegic patterns during walking.

Functional electrical stimulation (FES) is a common representative of the bottom-up peripheral stimulation method for stroke (10). FES has been used to improve foot drop after stroke since 1960 (11) and has been proven to alter circumduction hemiplegic gait patterns, considerably improve the step length and the maximum dorsiflexion and knee flexion angles, and increase the maximum muscle forces of both tibialis anterior and rectus femoris muscles (12). Moreover, the muscular structures of the tibialis anterior, rectus femoris, and gastrocnemius muscles are reversible with long-term FES use, as decreased echogenicity of the tibialis anterior muscle, accompanied by increased muscle size on the paretic side, was found (13). It is most likely that FES provides a different stimulation context for the excitability of the common peroneal nerve, which becomes more susceptible to overuse and fatigue, leading to a decrease in motor-evoked potential (MEP) parameters and motor plasticity (13). However, the decreased excitability of the motor cortex reflected by MEPs cannot prove that FES has a motor plasticity effect. Conversely, previous studies reported that FES did not simply increase the general excitability of the cortex and had specific effects on particular cortical neurons (14). Possibly attributable to a bimodal balance-recovery model, there are different patterns of neural reorganization, such as interhemispheric competition or ipsilateral vicariation, in the injured motor cortex, with different structural reserves (15). However, it remains uncertain how FES affects motor cortical plasticity. Although our previous study found that FES treatment could enhance brain functional connectivity and efficiency in hemiplegic patients, it did not address cortical activation patterns during the locomotor control process (16). As for the second purpose of our research, the present study aimed to explore the possible mechanism of FES in walking rehabilitation by monitoring the effect of FES-assisted walking on brain activation during hemiplegic walking using fNIRS.

## Methods

### Participants

Patients with post-stroke who satisfied the following criteria were included in this study: (i) diagnostic criteria for major cerebrovascular diseases in China formulated by the Chinese Society of Neurology, which were applied to diagnose and enroll the patients in this study; (ii) right hemiplegia in the first brain stroke; (iii) subcortical lesions confined to the left hemisphere; (iv) right hemiplegic lower limb function at Brunnstrom stage ≥IV; (v) ability to continuously walk for 10 m for 15 s independently or with the assistance of walking aids, such as ankle foot orthoses and custom-fit insoles for reducing foot drop; and (vi) absence of cognitive impairment and Mini-Mental State Examination (MMSE) score of ≥24 for middle school or higher education and MMSE score of ≥21 for elementary education and no education in order for patients to correctly execute the training test instructions (17). Moreover, the healthy individuals had to satisfy the following criteria: (i) no history of neurological, physical, or psychiatric impairment; (ii) MMSE score ≥24; and (iii) no insomnia, alcohol consumption, or medication usage in the last week. Individuals who had head skin damage or large scar areas, recently used sedatives, or consumed alcohol were excluded. Written informed consent was provided by each participant prior to study commencement, and the study was approved by the Ethics Committee of Xiamen Fifth Hospital (approval number: 2020-XMSDWYY-009).

A total of eight healthy individuals with right leg dominance (male patients, 75%; mean age, 40.06 ± 4.53 years) and six post-stroke patients with right hemiplegia (male patients, 86%; mean age, 44.41 ± 7.23 years; disease course, 5.21 ± 2.63 months) were recruited for this study. There were no significant differences in sex or age between the two groups (*p* > 0.05). All participants had normal cognitive functions, although the MMSE score of healthy individuals was significantly greater than that of hemiplegic patients (*p* = 0.01) (Table 1).

**Table 1:** Basic data of participants.

**Variable**	**Healthy participants**	**Post-stroke patients**
Age (years)	40.06 ± 4.53	44.41 ± 7.23
Time from stroke (months)	/	5.21 ± 2.63
Sex (males, %)	75%	86%
Stroke type (ischemic/hemorrhagic)	/	2/4
MMSE	28.37 ± 1.41	25.17 ± 1.47

Values are presented as mean ± SD or as frequency. MMSE, Mini-Mental State Examination.

### Experimental setup

A speed of 2 km/h was found to be a relatively appropriate treadmill speed, which was not too slow for healthy participants to feel comfortable walking while, at the same time, one to which the hemiplegic patients in this study were also able to adapt. The participants walked on a TecnoBody Digital Platform treadmill (ProKin 254P, TecnoBody, Italy) at a speed of 2 km/h while wearing comfortable clothing. Hemiplegic patients wore suspension measures to prevent falls; however, the suspension did not affect their weight. The participants kept their eyes straight and paid attention to a real-time moving image on the screen in front of them while walking. The participants' swinging limbs and torso during walking were displayed at the center of the screen. The walking range of both lower limbs and each foot weight were shown on the lower side of the screen. The participants were instructed to walk in a straight line, to avoid leaning toward one side of the treadmill track, and to keep their shoulders horizontal.

A four-channel FES therapeutic instrument (Model P2-9632, Guangzhou Fanke Medical Equipment Co., Ltd., China) was used. Four groups of muscles—namely, the tibialis anterior muscle, middle and lateral heads of the quadriceps femoris muscle, gastrocnemius muscle, and hamstring muscle—were marked in the movement at the muscle belly point and affixed by four sets of electrodes. The parameter settings were as follows: walking mode; biphasic square wave; frequency of 30 Hz; pulse width of 200 μs; 5-s walking cycle; and current intensity limited to the maximum tolerance of participants.

### fNIRS imaging

**Figure 1:**
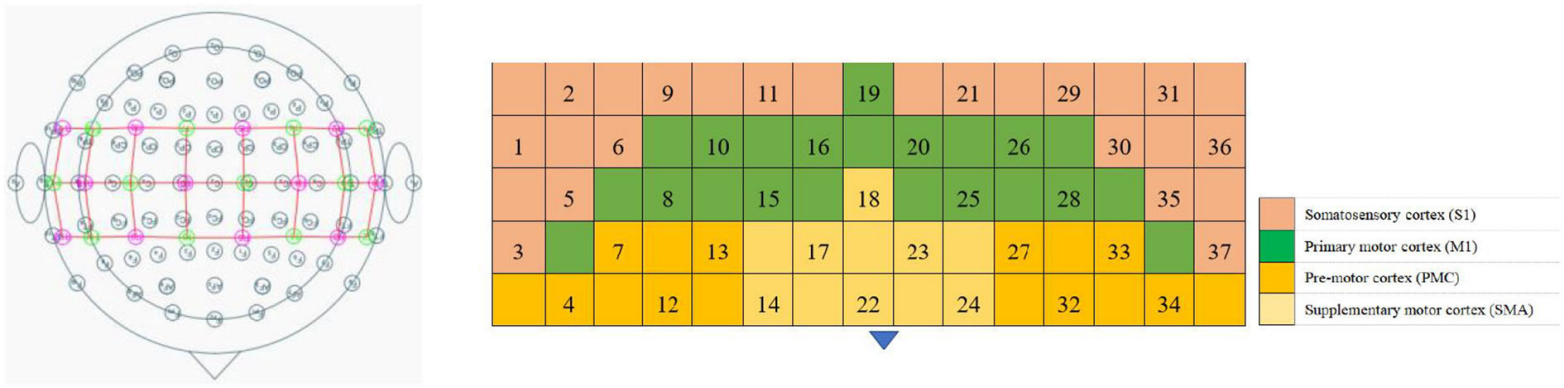
Schematic diagram of fNIRS channel registration with brain regions.

The fNIRS equipment (Model BS-3000, Wuhan Znion Technology Co., Ltd., Wuhan, China) used 858- and 764-nm wavelengths, and the light intensity data were sampled at 20 Hz. To normalize the fNIRS channels, we applied a 3D digitizer (Nirmap, Wuhan Znion Medical Technology Co., Ltd., Wuhan, China) to record the exact spatial coordinates of four reference points (central zero, nasion zero, AL, and RL) and 24 probes (12 sources and 12 detectors). Subsequently, 37 channels were converted to an estimated Montreal Neurological Institute space (18) by NIRS-SPM (19). Based on the Brodmann probabilistic atlas, all 37 channels were then divided into the following five cortical regions: premotor cortex (PMC), supplementary motor area (SMA), primary motor cortex (M1), and primary somatosensory cortex (S1), among which the M1 and S1 were collectively referred to as the sensorimotor cortex (SMC) (Figure 1 and Table 2).

**Table 2:** List of fNIRS channels corresponding to Brodmann partitions.

**Brodmann areas**	**Channel number**
Primary somatosensory cortex: BA 1, 2, 3	Ch03/05/06/09/11, ch21/29/30/35 /37Ch01/36
Supramarginal gyrus part of Wernicke's area: BA40	Ch02/31
Subcentral area: BA43	Ch3/37
Primary motor cortex: BA4	Ch08/10/15/16/19/20/25/26/28
Premotor cortex: BA6	Ch14/17/18/22/23/24
Supplementary motor cortex: BA6	Ch04/07/12/13/27/32/33/34

fNIRS, functional near-infrared spectroscopy.

### Experimental procedure

**Figure 2:**
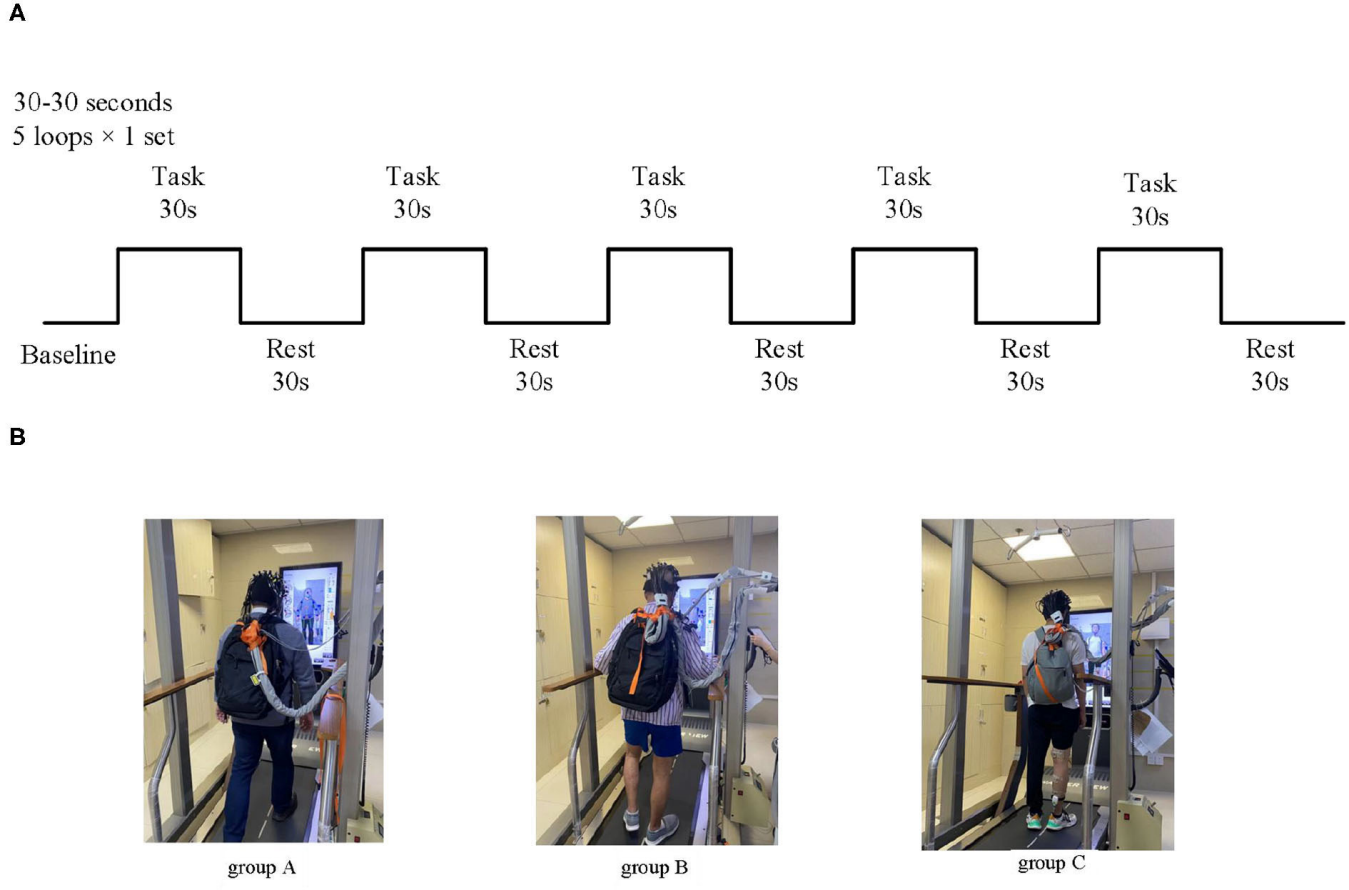
Experimental block design for walking modes at a consistent speed. **(A)** Treadmill walking and FES-assisted walking modes. **(B)** Schematic diagram of experimental grouping; Group A: healthy treadmill walking at a speed of 2 km/h; group B: hemiplegic treadmill walking at a speed of 2 km/h; and group C: hemiplegic treadmill walking with FES assistance at a speed of 2 km/h.

The whole test process was divided into two stages: preparation and task. During the preparation period, the participants rested in a standing position for 10 s. During the task period, upon hearing the command “walk,” the participants first stepped on the right leg and walked alternately for 30 s; when hearing the command “stop,” the participants stopped walking and rested in a standing position for 30 s. This process was repeated for five loops (Figure 2).

### Data analysis

Light intensity data were converted into changes in oxy-Hb and deoxy-Hb concentrations using a built-in function based on the modified Beer–Lambert law (20). Signal analysis was performed using the MATLAB 2014b toolbox (MathWorks, Inc., Natick, MA, USA).

#### Cortical activation

**Figure 3:**
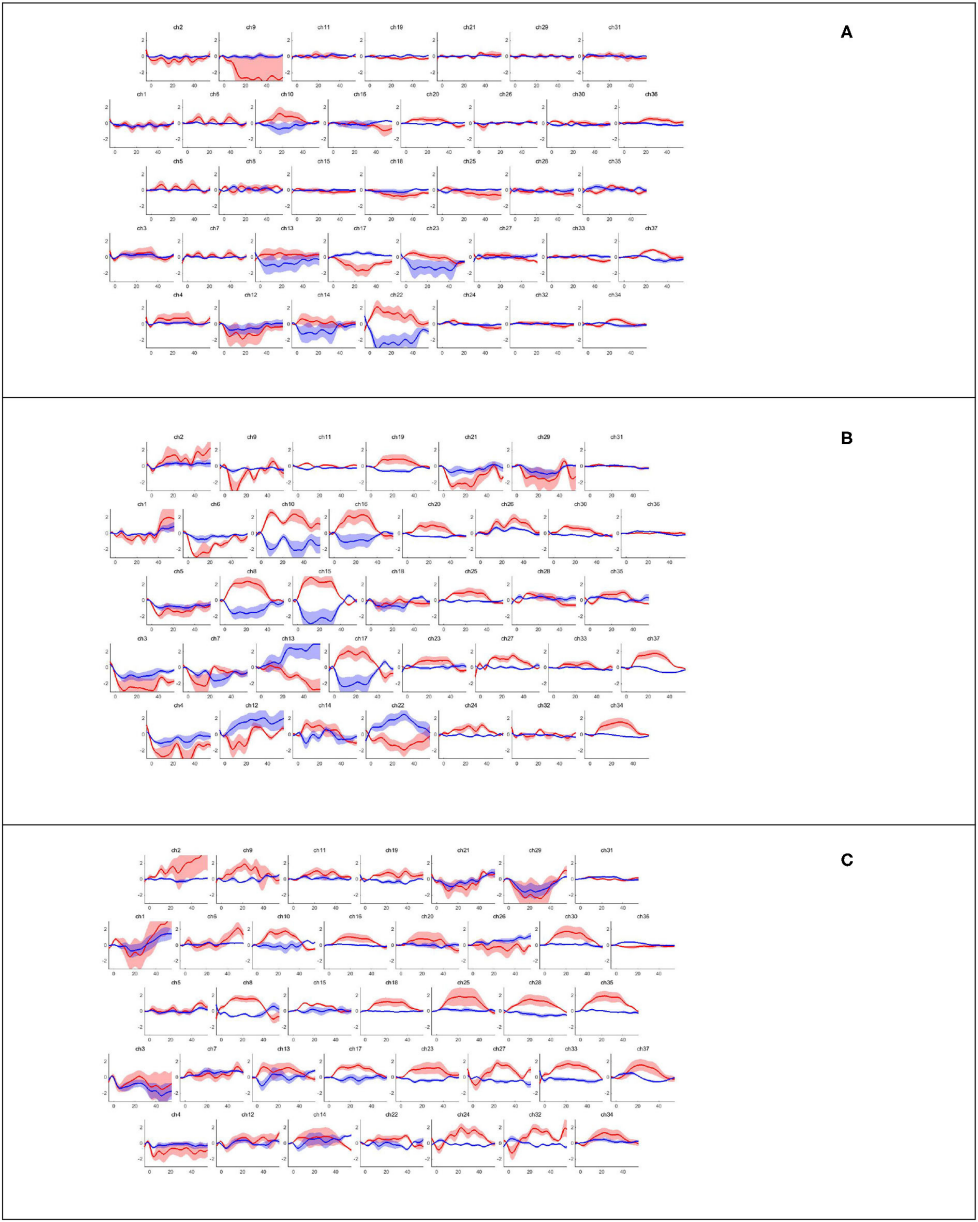
Hemodynamic response corresponding to walking task-rest phase by channels. Block averaging of the hemodynamic response (hmrBlockAvg) was designed within the time range of −5 s to 55 s; the baseline hmr level was 5 s before the task began. The oxy-Hb and deoxy-Hb concentrations are indicated by the red and blue lines, respectively. **(A)** Healthy treadmill walking, **(B)** hemiplegic treadmill walking, and **(C)** hemiplegic treadmill walking with FES assistance.

NIRS-KIT (registration number for software copyright protection: 2019SR1299168; Beijing Normal University, China) (21) can analyze hemodynamic changes in oxy-Hb and deoxy-Hb concentrations from raw light intensity time series using general linear models. The brain's hemodynamic responses (hmr) to a task condition, including the changes in oxy-Hb and deoxy-Hb concentrations in brain regions (Figure 3) in our study, are assumed to be linearly additive and consistent across trials. This pipeline of task activation detection comprised common and necessary processing steps for fNIRS signal analysis, such as data preparation, quality control, preprocessing, individual-level analysis, group-level statistics, and visualization of results. In the preprocessing step, which consisted of several parts including detrending, the temporal derivative distribution repair (TDDR) motion correction method was adopted for TDDR (22), with bandpass filtering (0.001 to 0.08 Hz) by a third-order infinite impulse response filter. Oxy-Hb levels increased at 3–5 s after the onset of each task, reached a plateau at 10 s, and decreased at 3–5 s after the end of each task; based on this, we acquired images depicting the average from Δoxy-Hb changes, which were obtained *via* block-averaging of five tasks within the time range of 0–35 s minus the baseline hmr level of −5 s before each task began. The MATLAB customized processing method was used to process fNIRS data. A general linear model was used to detect task activation in the individual-level analysis of the task's fNIRS. In group-level analyses, a one-sample *t*-test was used to test whether the β indices were significantly different (from a given value, e.g., 0) between the healthy and patient groups. The two-sample *t*-test was used to examine whether the β indices in the healthy and patient groups were significantly different from each other. The paired *t*-test was used to determine whether the β indices in the treadmill and FES-assisted walking groups were significantly different from each other. The false discovery rate was used to correct for multiple *t*-testing of fNIRS data in each channel (23). Before the correction, the statistical significance level for all comparisons was set at *p* < 0.05 (two-sided). Channel-wise 2D visualization was used to show the task-design fNIRS group-level analysis indices (beta value or contrast value).

#### Laterality index

The laterality index (LI) was used to evaluate the asymmetry in each region's amount of activation. LI values ranged from −1 to 1; a positive value (0 to 1) indicated right-dominant activation, whereas a negative value (−1 to 0) indicated left-lateralized activation. The oxy-Hb change activation in the brain region of interest was compared using SPSS software version 25.0 (Statistical Product Service Solutions, IBM Corp., USA). The LI was defined as [Δoxy-Hb in the right (unaffected) hemisphere – Δoxy-Hb in the left (affected) hemisphere]/[Δoxy-Hb in the right (affected) hemisphere + Δoxy-Hb in the unaffected hemisphere]; in brief, (R – L) / (R + L) (24). Δoxy-Hb changes were selected in the same time window as the above cortical activation. For the comparison of LI in group-level analysis, we performed a two-way repeated-measures analysis of variance (ANOVA) to test the interactions (3 × 4: type of gait [healthy gait, hemiplegic gait, and hemiplegic gait with FES assistance] × site of cortical regions [S1, M1, SMA, and PMC]). Fisher's least significant difference test was used as a *post hoc* test. Statistical significance was set at a *p*-value of < 0.05 for all comparisons.

## Results

### Cortical mapping of the healthy and hemiplegic gait

**Figure 4:**
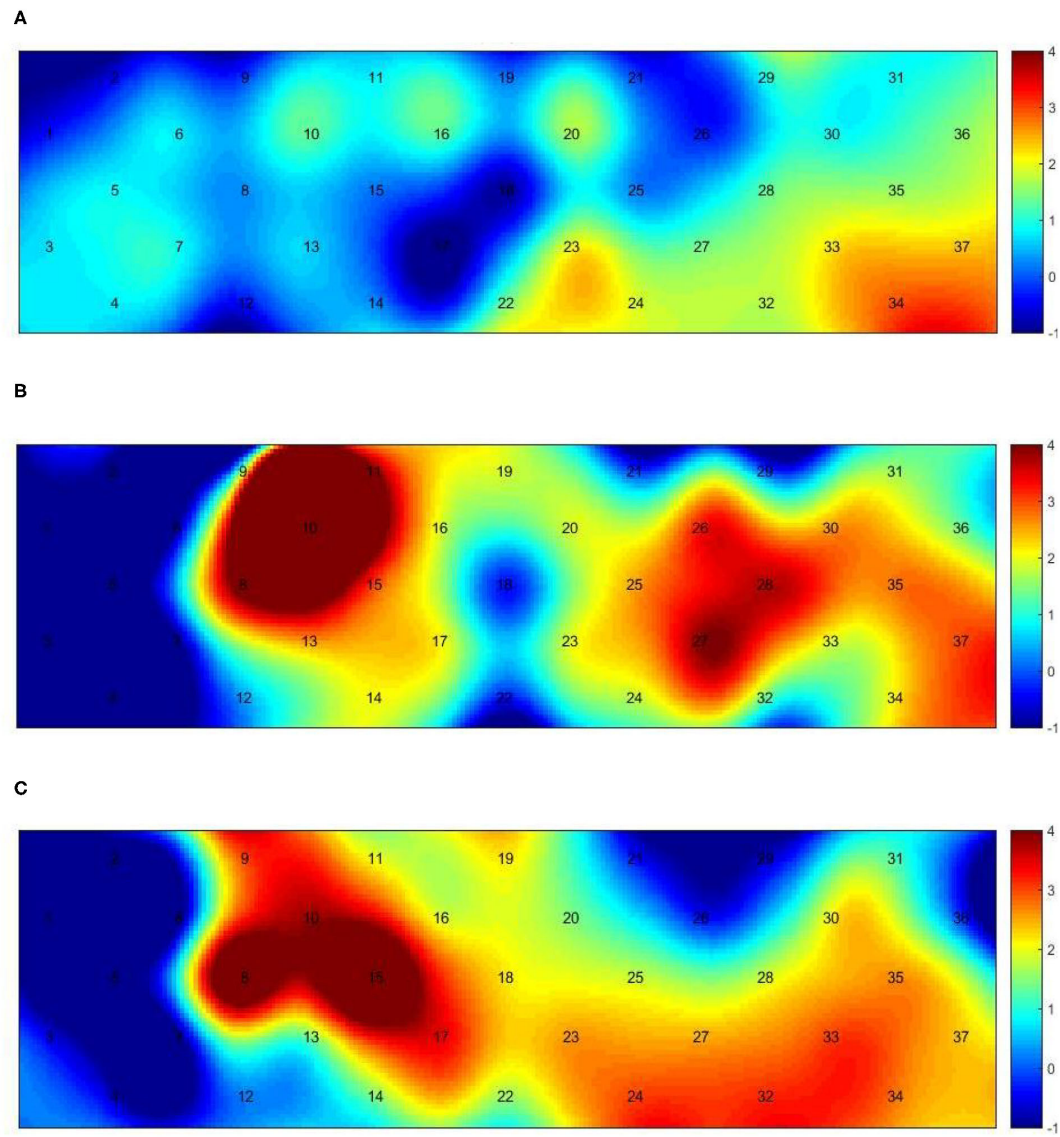
Group-level channel activation t-map in the gait task (*p* < 0.05, uncorrected). **(A)** Healthy treadmill walking, **(B)** hemiplegic treadmill walking, and **(C)** hemiplegic treadmill walking with FES assistance.

**Figure 5:**
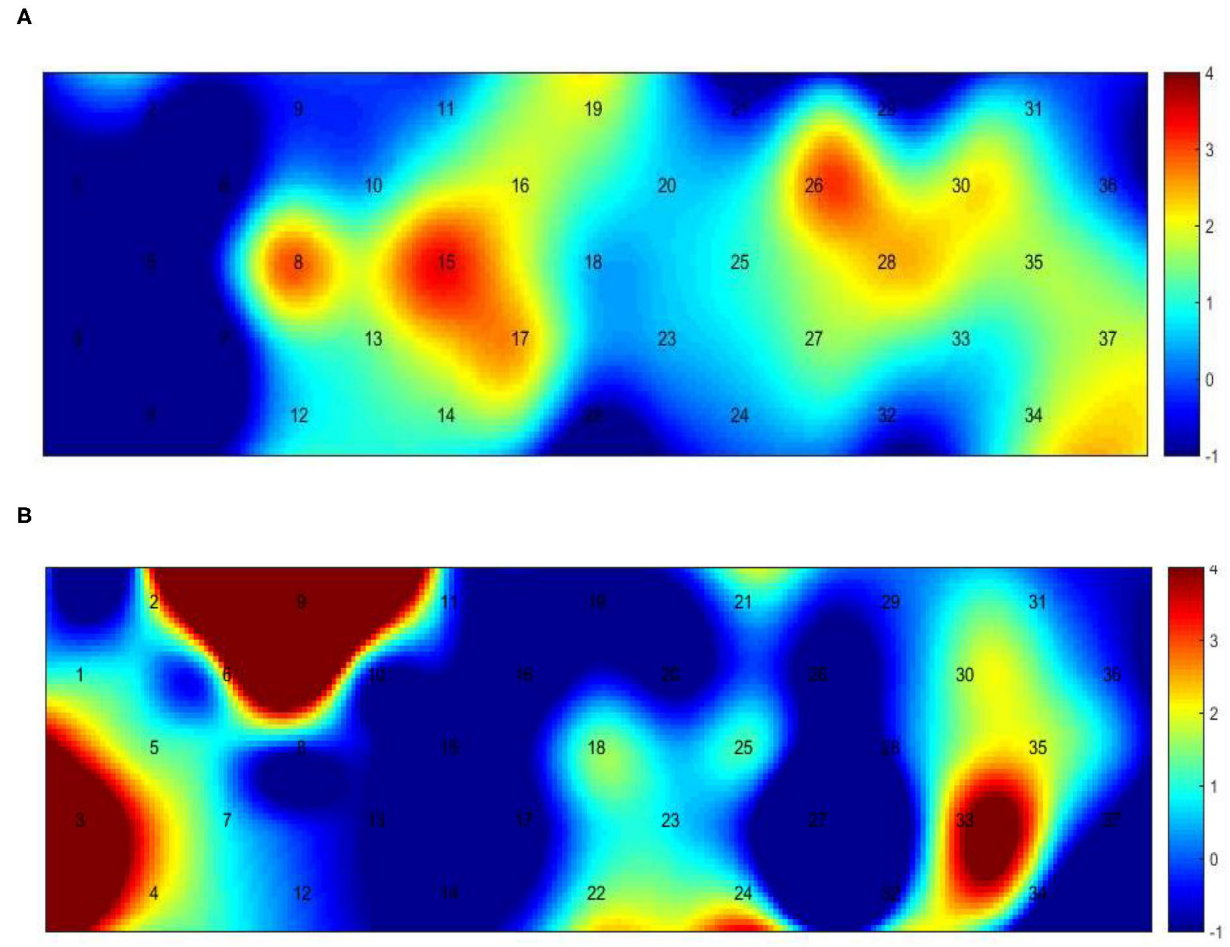
Between-group variance of channel activation t-map in the gait task (*p* < 0.05, uncorrected). **(A)** The variance of healthy and hemiplegic treadmill walking. **(B)** The variance of hemiplegic treadmill walking with or without FES assistance.

Figure 3 presents the trends of hemodynamic changes in oxy-Hb and deoxy-Hb concentrations across all channels under three conditions, which showed several obvious characteristics. Figures 4A, B and Table 3 show the cortical activation patterns during healthy and hemiplegic gait on a treadmill at the group level. Almost no activation in the bilateral M1 and apparent activation in the left PMC (ch33/34) and S1 (ch37) were observed during walking in healthy individuals (Figure 4A). There was more activation in the SMC, including the S1 and M1 (ch8/10/11/15), in the unaffected hemisphere than in the affected hemisphere (ch25/26/28/37). More PMC activation (ch34) in the affected hemisphere was noted during hemiplegic gait (Figure 4B). In addition, group differences in cortical activation were observed. The M1 and S1 regions (ch8/15 and ch26/28) in both hemispheres were more activated, and SMA (ch17) and PMC (ch34) activation appeared to be more prominent in hemiplegic patients than in healthy individuals (Figure 5A and Table 3). Generally, the most characteristic finding was the greater locomotor network activation of the SMC-PMC-SMA during the hemiplegic gait than that during the healthy gait.

**Table 3:** List of group-level channel activation in the task (t-value, *p* < 0.05).

	**Left**	**Right**
Healthy	Ch33 (2.496, 0.041), ch34 (3.424, 0.011), ch37 (2.514, 0.040)	-
Hemiplegia	Ch25 (2.678, 0.044), ch26 (3.267, 0.022), ch27 (3.983, 0.010), ch28 (3.670, 0.014), ch33 (2.726, 0.041), ch34 (2.843, 0.036), ch37 (3.334, 0.021)	Ch8 (3.800, 0.013), ch10 (14.052, 0.000), ch11 (2.900, 0.034), ch15 (3.188, 0.024)
Hemiplegia with FES	Ch23 (2.667, 0.045), ch24 (3.324, 0.021), ch27 (2.685, 0.044), ch32 (3.379, 0.020), ch33 (3.199, 0.024)	Ch8 (5.003, 0.004), ch9 (3.349, 0.020), ch10 (3.692, 0.014), ch15 (5.726, 0.002), ch17 (3.334, 0.021)
Group differences in cortical activation between healthy and hemiplegic gait	Ch26 (3.030, 0.010), ch28 (2.457, 0.030), ch34 (2.294, 0.041)	Ch8 (2.879, 0.014), ch15 (3.402, 0.005), ch17 (2.515, 0.027)
Group differences in cortical activation between hemiplegic gait with FES and hemiplegic gait without FES	Ch24 (3.229, 0.023), ch33 (4.971, 0.004)	Ch3 (11.342, 0.000), ch9 (61.251, 0.000)

FES, functional electrical stimulation.

Cortical activation mapping during FES-assisted hemiplegic walking at the group level is shown in Figure 4C and Table 3. The SMC (ch9, ch8/10/15) was significantly activated in the unaffected hemisphere, whereas the SMA and PMC (ch17/23/24 and ch27/32/33) were activated in the affected hemisphere. Group differences in cortical activation were also apparent between hemiplegic walking with and without FES assistance. Increased oxy-Hb changes were observed in the S1 (ch3/9) on the unaffected side and in the SMA (ch24) and PMC (ch33) on the affected side during hemiplegic walking with FES, as compared to that without FES (Figure 5B and Table 3). Generally, the most characteristic finding was SMA and PMC activation in the affected hemisphere, but not SMC activation, which was a noticeable change induced by FES.

### Cortical activation symmetry during healthy and hemiplegic gait

**Figure 6:**
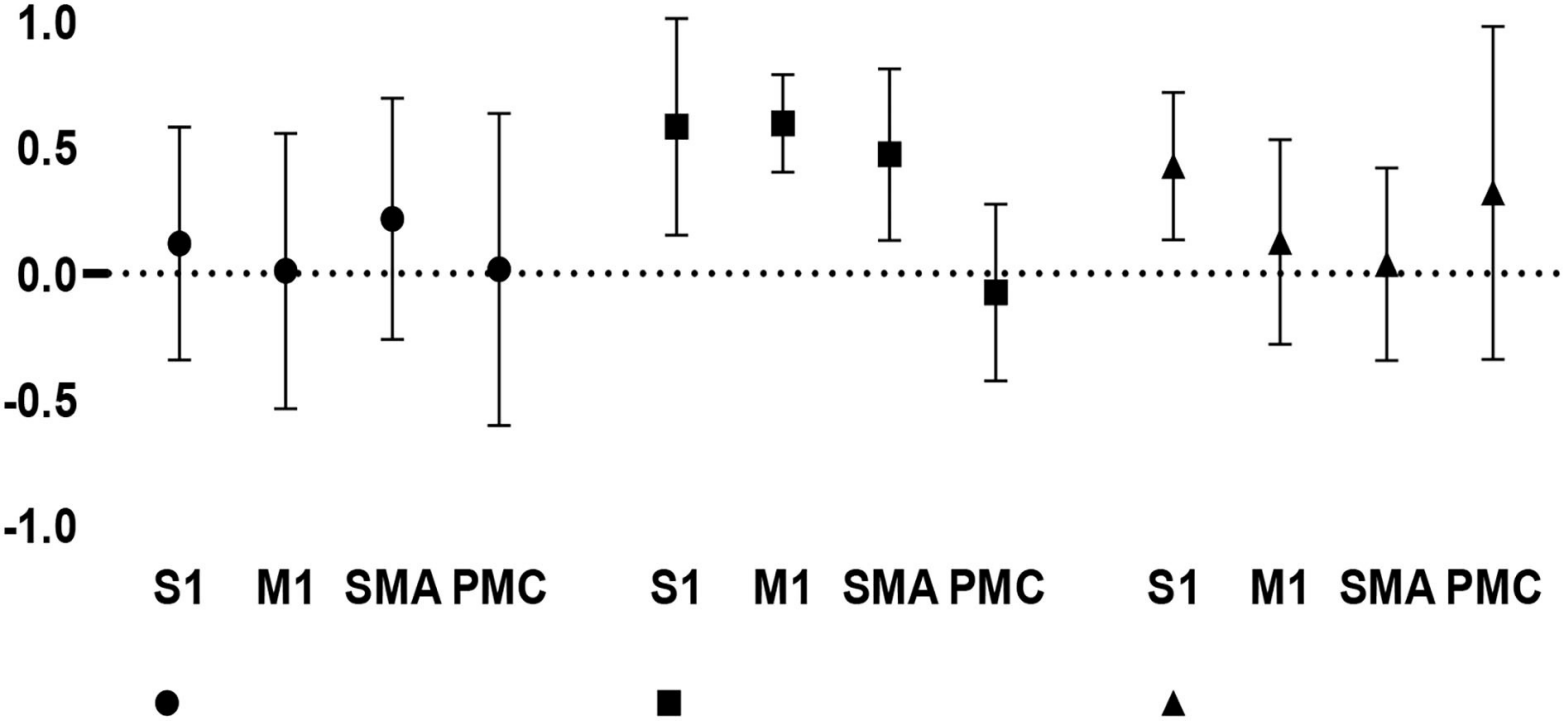
Changes in interhemispheric regional activation asymmetry during gait in the three groups, as measured by LI.

The LI values (mean ± SD) were 0.120 ± 0.463 in the S1, 0.011 ± 0.547 in the M1, 0.218 ± 0.479 in the SMA, and 0.017 ± 0.620 in the PMC, with a total value of 0.092 ± 0.530 for healthy walking. The LI values (mean ± SD) were 0.584 ± 0.513 in the S1, 0.597 ± 0.184 in the M1, 0.473 ± 0.324 in the SMA, and −0.075 ± 0.335 in the PMC, with a total value of 0.395 ± 0.367 for hemiplegic walking. The LI values (mean ± SD) were 0.428 ± 0.279 in the S1, 0.126 ± 0.387 in the M1, 0.038 ± 0.364 in the SMA, and 0.321 ± 0.630 in PMC, with a total value of 0.228 ± 0.435 for hemiplegic walking with FES assistance. The LI in Figure 6 shows the changes in interhemispheric asymmetry of regional activation during walking among the various groups.

Repeated-measures ANOVA for the LI indicated no significant gait × cortical region interaction (F_[6, 68]_ = 1.578, *p* = 0.167). A significant main effect for the type of gait (F_[2, 68]_ = 3.172, *p* = 0.048) was found; in contrast, there was no significant main effect for the site of cortical regions (F_[3, 68]_ = 1.386, *p* = 0.254). The *post hoc* (least significant difference) test showed that the total positive LI value was greater during hemiplegic gait than during healthy gait (*p* = 0.014), suggesting that the hemiplegic gait induced a relatively greater unaffected (right-dominant) activation than the healthy gait during TW. Moreover, there was a significant difference in the S1 (t_[12]_ = 2.337, *p* = 0.037) between hemiplegic gait (0.584 ± 0.513) and healthy gait (0.120 ± 0.463) as well as a significant difference in the M1 (t_[12]_ = 2.420, *p* = 0.032) between the hemiplegic gait (0.597 ± 0.184) and healthy gait (0.011 ± 0.547), suggesting that hemiplegia induced a relatively greater bilateral interhemispheric asymmetry in the S1 and M1 in the unaffected (right) hemisphere. Conversely, no significant differences in LI values were detected between the other groups (*p* > 0.05), indicating no significant lateralized activation in healthy or FES-assisted hemiplegic walking.

Furthermore, within the hemiplegic group, the LI values were significantly greater in the S1 (0.584 ± 0.513), M1 (0.597 ± 0.184), and SMA (0.473 ± 0.324) than in the PMC (−0.075 ± 0.335) (F_[3, 20]_ = 9.586, *p* < 0.001). This suggests that, in the phenomenon of asymmetric cortical activation, the M1, S1, and SMA were predominantly activated in the unaffected hemisphere, whereas the PMC was predominantly activated in the affected hemisphere during hemiplegic walking. No significant difference in LI values was noted in the healthy and FES-assisted hemiplegic walking groups (*p* > 0.05).

## Discussion

The present fNIRS study examined cortical activation responses in healthy individuals and hemiplegic patients during treadmill and FES walking at a speed of 2 km/h. In this study, we found that hemiplegic patients exhibited more SMC-SMA-PMC cortical activation during the walking process than healthy individuals. Previous studies have supported the critical role of the locomotor control network in walking. Using fNIRS mapping, Kim et al. demonstrated increased SMC, PMC, and SMA activation during conventional stepping walking, TW, and robot-assisted walking in 14 healthy individuals (25). Miyai et al. found that walking activities on a treadmill were bilaterally associated with increased oxy-Hb levels in the medial primary SMC and SMA in eight healthy individuals (26). Stroke leads to damage to the motor cortices and their descending corticospinal tracts and subsequent muscle weakness (27), so we speculate that patients with post-stroke may need to recruit more locomotor cortical networks in gait locomotion control, in which some prominent activation in SMC-SMA-PMC is involved in the hemiplegic walking process.

We observed the obvious feature of almost no activation of the bilateral M1 in healthy individuals during TW. Koenraadt et al. also proved a similar phenomenon in 11 healthy participants; the sensorimotor cortex showed no activation change during walking, whereas SMA showed mainly increased activation before the start of TW tasks (8). The lack of M1/S1 activation during gait may suggest that ongoing gait control mainly relies on subcortical automatisms, such as central pattern generators (28) because it has long been known from animal studies that the M1 is not essential for automated unperturbed gait (29). Our negative findings for the M1 were in line with the results obtained in fNIRS studies by Koen et al. during TW at a speed of 3 km/h (8), by Suzuki et al. at speeds of 3 and 5 km/h (30), by Presacco et al. (31) using EEG at a maximum speed of 2.4 km/h, and by Nordin et al. (32) at different levels of gait speeds (1.8, 3.6, 5.4, and 7.2 km/h), wherein spectral power fluctuations showed reduced left and right sensorimotor alpha and beta EEG waves across the healthy gait cycle. Thus, because the 2 km/h gait speed in the present study lies within the slow speed category, our results contribute evidence of the fact that locomotor speed is largely controlled subcortically (33, 34). Hence, relying on subcortical structures without excessive recruitment of SMC activation is sufficient to support automated movements in healthy individuals.

However, during TW in hemiplegic patients, asymmetric cortical activation was observed as an important feature. The fNIRS mapping showed more SMC activation in the unaffected hemisphere than in the affected hemisphere and more PMC activation in the affected hemisphere. Furthermore, the LI expressing bilateral interhemispheric asymmetry revealed that hemiplegic walking induced relatively greater activation in the unaffected (right-dominant) hemisphere. The LI regarding inter-regional asymmetry revealed SMC and SMA cortex-lateralized activation in the unaffected hemisphere and PMC-lateralized activation in the affected hemisphere during hemiplegic walking. This was in line with the reports that hemiparetic gait was associated with greater increases in oxy-Hb levels in the medial SMC of the unaffected hemisphere than that in the affected hemisphere (3, 9, 35), possibly related to the inhibitory mechanism of interhemispheric competition.

Obviously, during hemiplegic walking with FES, the noticeable characteristic was SMA and PMC activation in the affected hemisphere, rather than the SMC in the unaffected hemisphere, which seems to be a change induced by FES assistance (Figure 3C). This was similar to the finding that FES-assisted walking training could reduce the MEP latency time of the tibialis anterior muscle and improve the excitability of the corresponding motor cortex (14, 16). Owing to the influence of movement noise, there are few studies on real-time brain functional imaging during FES-assisted walking training. Zheng et al. evaluated the four-channel FES training effect in patients with stroke using fMRI and found that FES increased structural and functional reorganization around the lesion on the affected side (36). Our research focused on the salient activation of the SMA and PMC of the affected hemisphere during hemiplegic walking with FES. Previous research has supported the function of SMA and PMC in the initiation of complex motor activities and postural control (37). In particular, PMC is highly involved in both cognitive and motor dual-task challenges during both healthy and post-stroke adult walking (38). The increased SMA and PMC activation reflects the demand for adaptive locomotion control, compensation, or reorganization of cortical networks, which may represent the intrinsic mechanism of FES that has been proven to promote the rehabilitation of walking and balance functions in patients (39).

Interestingly, there was no significant difference in LI values for bilateral interhemispheric and inter-regional symmetry between healthy walking and FES-assisted hemiplegic walking. It seems that FES may help hemiplegic walking recover the balance in cortical activation. The following reports can also be indicative. Asymmetric cortical excitability has been found in hemiplegic patients, wherein the affected hemisphere had a higher stimulation threshold and lower MEPs than the unaffected hemisphere (40). Moreover, interhemispheric inhibition imbalance aggravated bilateral MEP asymmetry and led to the disappearance of MEPs in the affected motor area (41). As rehabilitation progressed, increased PMC activation was found in the affected side after 2 months of TW training, and the asymmetry of bilateral SMC activation was improved (35). Song et al. reported that cortical activation in patients with stroke was lower in the affected hemisphere than in the unaffected one, while asymmetric activation performance was improved after 3 weeks of robot-assisted gait training (42). Similarly, our research also found asymmetric cortical activation during hemiplegic walking, improved bilateral activation during FES-assisted hemiplegic walking in patients with post-stroke, and speculated that a new interhemispheric balance may be re-established by activating specific brain regions in SMA and PMC on the affected side. FES could be used as a potentially powerful therapeutic strategy, which may play an important role in motor recovery after a stroke. This is worthy of further clinical research and verification.

This study has some limitations. First, the study focused on motor-related cortical regions, including SMC, SMA, and PMC, while the role of the prefrontal cortex (PFC) in gait control was not investigated. PFC activation occurs during normal gait, whereas higher PFC activation occurs during Ekso-assisted walking in patients with stroke (43); in addition, PFC is also activated for longer during precise walking (8). This suggests that more prefrontal cortical metabolism is involved in locomotion control with more complex tasks and when extra attention is required, as in patients with post-stroke and healthy older age people during walking (44, 45). Second, the TW speed of 2 km/h was used in this study to adapt to hemiplegic patients and to ensure safety. Thus, the effect of different walking speeds was not taken into account on brain cortical activation. The literature reports that, as walking speed increases, multiple locomotor network activations are observed and the activation power spectrum increases (25). Conversely, some studies have found that higher walking speeds cause more motor network activation during TW at 3 and 5 km/h (8, 30). These factors should be explored in future locomotor gait control studies.

In conclusion, the present fNIRS study showed that there was more locomotor cortical activation of the SMC-PMC-SMA during hemiplegic gait than during healthy gait. Moreover, in hemiplegic gait, there was more SMA and PMC activation in the affected hemisphere during the FES-assisted task than during the non-FES-assisted task. The LI indicated asymmetric cortical activation during hemiplegic gait, inducing relatively greater activation in the unaffected (right) hemisphere during hemiplegic gait than during healthy walking. Furthermore, SMC and SMA were predominantly activated in the unaffected hemisphere, with PMC being predominantly activated in the affected hemisphere during hemiplegic walking. One obvious feature was that almost no activation in the bilateral M1 was noted during healthy walking, whereas more SMC-SMA-PMC cortical activation was involved in hemiplegic walking, possibly related to the M1. This suggests that M1 may not be essential for automated unperturbed gait in healthy individuals and that patients with post-stroke would need to recruit more locomotor networks to control walking. Asymmetric cortical activation was another important feature of hemiplegic walking, possibly related to the inhibitory mechanism of interhemispheric competition. Increased SMA and PMC activation in the affected hemisphere reflected the demand for adaptive locomotion control, compensation, and reorganization of cortical networks. In this regard, FES-assisted walking appears to increase the activation effect, which is worth verifying through additional research.

## Data availability statement

The original contributions presented in the study are included in the article/supplementary material, further inquiries can be directed to the corresponding authors.

## Ethics statement

The studies involving human participants were reviewed and approved by the Ethics Committee of Xiamen Fifth Hospital, 2020-XMSDWYY-009. The patients/participants provided their written informed consent to participate in this study. Written informed consent was obtained from the individual(s) for the publication of any potentially identifiable images or data included in this article.

## Author contributions

XKH designed the study. LL, GY, XL, and QQS conducted the study, including patient recruitment and data collection. XKH and SJC contributed to the data analysis. SJC prepared the manuscript draft, with important intellectual input from XKH. All authors approved the final manuscript.
